# Supplementary material for: Splice-Junction-Based Mapping of Alternative Isoforms in the Human Proteome
Source: Cell Rep. Author manuscript; Available in PMC 2020 Jan 15. (PMC6961840; doi:10.1016/j.celrep.2019.11.026)

A

sp|Q15366|PCBP2_HUMAN|ENSG00000197111|A3SS2|2045|chr12|53467293|53468832|+0|r12|T1
LTKLHQLAMQQSHFPMTHGNTGFSGLDASAQTTSHE q value: 0.00020194 Tr_novel:TRUE RefSeq_Novel:TRUE
Search result spec prec mz: 977.9747 Actual spec prec mz: 977.97466
Fragments matched per AA: 1.39 Proportion of top 20 peaks matched: 0.25

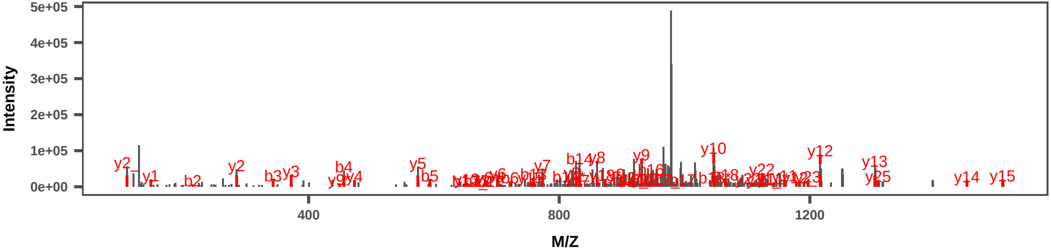

B

Scatterplot of predicted elution time
Fitting R2: 0.65
Novel peptide residual Z score: −0.675
Number of peptides: 1395

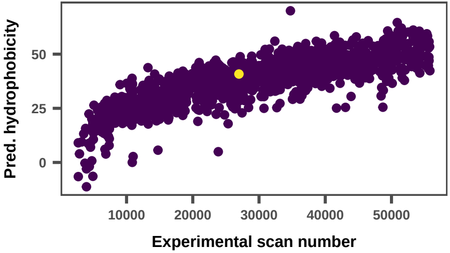

C

Distributions of residuals from best−fit line
of predicted RT vs Expt. scan number
Line: Z score of novel peptide
Z: −0.675

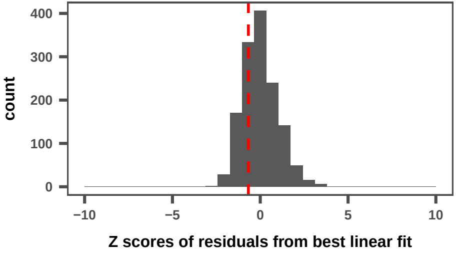

Supplement: 2 [file NIHMS1546469-supplement-2.zip › DF1/PXD009737/TestisGluC/TestisGluC_2_PCBP2_LTKLHQLAMQQSHFPMTHGNTGFSGLDASAQTTSHE.pdf]
